# Linear Endobronchial Ultrasound in the Era of Personalized Lung Cancer Diagnostics—A Technical Review

**DOI:** 10.3390/jcm10235646

**Published:** 2021-11-30

**Authors:** Filiz Oezkan, Stephan Eisenmann, Kaid Darwiche, Asmae Gassa, David P. Carbone, Robert E. Merritt, Peter J. Kneuertz

**Affiliations:** 1 Comprehensive Cancer Center, Division of Medical Oncology, The Ohio State University, Columbus, OH 43210, USA; David.Carbone@osumc.edu; 2 Department of Pulmonary Medicine, Section of Interventional Pneumology, Ruhrlandklinik-University Hospital Essen, University of Duisburg-Essen, 45239 Essen, Germany; Kaid.Darwiche@rlk.uk-essen.de; 3 Fifth Department of Internal Medicine, Faculty of University Heidelberg, University Medicine Mannheim, Theodor-Kutzer-Ufer 1-3, 68167 Mannheim, Germany; 4 German Cancer Research Center, A420 Research Group, 69120 Heidelberg, Germany; 5 Department of Pneumology, University Hospital of Martin Luther University, 06108 Halle, Germany; Stephan.Eisenmann@uk-halle.de; 6 Heart Center, Department of Cardiothoracic Surgery, Faculty of Medicine, University Hospital of Cologne, Kerpener Straße 62, 50937 Cologne, Germany; Asmae.Gasssa@uk-koeln.de; 7 Comprehensive Cancer Center, Division of Thoracic Surgery, Department of Surgery, The Ohio State University, Columbus, OH 43210, USA; Robert.Merritt@osumc.edu (R.E.M.); Peter.Kneuertz@osumc.edu (P.J.K.)

**Keywords:** lung cancer, endobronchial ultrasound, personalized lung cancer management

## Abstract

Major advances in molecular profiling for available targeted treatments and immunotherapy for lung cancer have significantly increased the complexity of tissue-based diagnostics. Endobronchial ultrasound-guided transbronchial needle aspirations (EBUS-TBNA) are commonly performed for diagnostic biopsies and lymph node staging. EBUS-TBNA has increasingly become one of the main sources of tumor cells for molecular analyses. As a result, there is a growing need for high quality EBUS-TBNA samples with adequate cellularity. This has increased the technical demands of the procedure and has created additional challenges, many of which are not addressed in the current EBUS guidelines. This review provides an overview of current evidence on the technical aspects of EBUS-TBNA in light of comprehensive sample processing for personalized lung cancer management. These include sonographic lymph node characterization, optimal needle choice, suction biopsy technique, and the role of rapid on-site evaluation. Attention to these technical details will be important to maximize the throughput of EBUS-TBNA biopsies for molecular testing.

## 1. Introduction

Linear endobronchial ultrasound (EBUS) is a well-established diagnostic tool for the workup and staging of lung cancer [1,2]. The diagnostic accuracy of EBUS for mediastinal staging has been shown to be as high as cervical mediastinoscopy and has demonstrated a favorable safety profile [3,4,5,6,7,8,9]. Over the past decade, the role of EBUS in the management of lung cancer has continued to evolve, concurrent with the major advances in the molecular profiling and available treatments. The management of NSCLC now requires multiple molecular tests to guide the treatment strategy for an increasing number of targeted agents [10,11]. In addition, immunologic testing and research are developing rapidly. While pembrolizumab and nivolumab were only approved by the US Food and Drug Administration (FDA) for second-line treatment in March 2016, several immunotherapies targeting the anti-programmed death receptor (PD)-1 and its ligand (PD-L1) have now been approved by the FDA for broader indications and have been expanded to first-line indications [12,13,14,15]. Targeted therapeutics and immunotherapies are also increasingly being considered for the management of earlier stage lung cancer.

Endobronchial ultrasound-guided transbronchial needle aspirations (EBUS-TBNAs) are becoming one of the main sources for tissue-based biomarker analysis. Time is of the essence for the management of lung cancer, and there should be a close partnership between the pulmonary, surgical, and medical specialties responsible for the management of lung cancer. Ideally, EBUS samples can be simultaneously sent by the practitioner for diagnosis, staging, and molecular analysis so that these data will be available to the medical oncologist at the first visit rather than being ordered post hoc, further delaying therapeutic decision making. To accomplish these goals, there is a growing need for high quality EBUS-TBNA tissue samples, which has created additional challenges and has increased technical demands, many of which are not reflected in the current EBUS guidelines. In this review, we sought to review technical aspects of EBUS as they relate to lung cancer diagnostics and treatment in the current era of personalized cancer care.

## 2. Sonographic Characteristics of Lymph Node Metastases

The identification and characterization of lymph nodes by EBUS is the first step in the selection of lymph nodes for biopsy, which is paramount considering the importance of accurate mediastinal staging. Ultrasonographic features to distinguish potentially metastatic lymph nodes from likely benign lymph nodes were first described by Fujiwara and colleagues in 2010 [16]. They retrospectively analyzed 1061 lymph nodes in 487 patients and showed that a round shape, which was defined as a ratio of the long to the short axis of <1.5, distinct margin, heterogeneous echogenicity, and the presence of a coagulation necrosis sign, a hypoechoic area within the lymph node without blood flow measured via Doppler, were independent predictors of lymph node malignancy. Consequentially, 96% of the lymph nodes that were negative for all four criteria were proven to be histologically benign [16]. A more detailed predictive score was developed by Schmid-Bindert and colleagues in 2012, which was based on six sonographic features: round shape, distinct margin, heterogeneous echogenicity, absence of central hilar sign (CHS), short axis >1 cm, and color power Doppler index (CPDI) of grade 2 or 3. The observed probability of lymph node malignancy was 80% when all six criteria were positive [17]. In 2015, Wang and colleagues published the utility of additional criteria, which included matting, calcification, and distinct vascular patterns, to distinguish between malignant and benign lymph nodes [18]. The highest specificity was reported for matting (96.59%), and the highest sensitivity was reported for the absence of a CHS (91.72%). The authors proposed an aggregate score system, with a 97.16% sensitivity for the detection of a malignant lymph nodes in the presence of matting, round lymph node shape, nonhilar perfusion, and absence of CHS [18]. In a review article written by Hylton et al. in 2018, CHS and heterogenous echogeneity were interpreted as the strongest predictors of malignant lymph nodes [16]. Considering that up to 42.14% of EBUS-TBNA samples are deemed insufficient for pathological assessment, the authors concluded that using these criteria, the absence of sonographic malignancy features would render repeat endobronchial ultrasound biopsies or surgical lymph node staging unnecessary [19,20]. While most of these criteria were derived from retrospective studies, a more recent prospective study included 300 lymph nodes from 140 patients analyzed in 7 Canadian centers. The four features of short axis length >1 cm, the absence of central hilar structure, <50% well-defined margins, and central necrosis were predictive of malignancy, whereas the echogenicity and shape (round versus oval), two features that have been widely used in other studies, were not predictive. On a multivariate model, size, margins, CHS, and central necrosis were the strongest predictors of malignancy. The combination of all four features reached a specificity of 99.59% [21].

**Figure 1:**
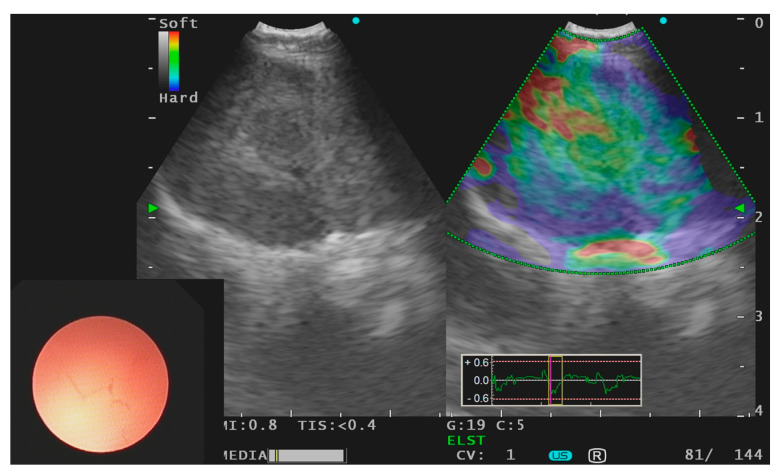
Endobronchial ultrasound using linear probe with use of elastography of a subcarinal lymph node harboring primary lung adenocarcinoma.

While the most widely used ultrasonographic features to distinguish malignant from benign lymph nodes remains the size (short axis of >1 cm), smaller lymph nodes may still harbor malignancy. A systematic sampling of mediastinal, hilar, and interlobar lymph nodes should therefore be performed even in light of false negative rates of radiologic and nuclear staging [22,23]. For the most accurate lymph node staging, all nodes measuring >0.5 cm on the short axis should be sampled starting at the N3 position and then at the N2 nodes and finally the N1 nodes to avoid tumor cell seeding. In addition, combining EBUS with endoscopic esophageal ultrasound investigation using the same EBUS bronchoscope (EUS-B) increases the sensitivity by 9% compared to a targeted EBUS procedure [24]. The sensitivity and the negative predictive value may be further increased by endobronchial elastography, which can be used to visualize the elasticity of the lymphoid tissue [25]. The characteristic heterogenous pattern of a tumor involved the lymph nodes is illustrated in Figure 1.

## 3. Biopsy Technique

**Figure 2:**
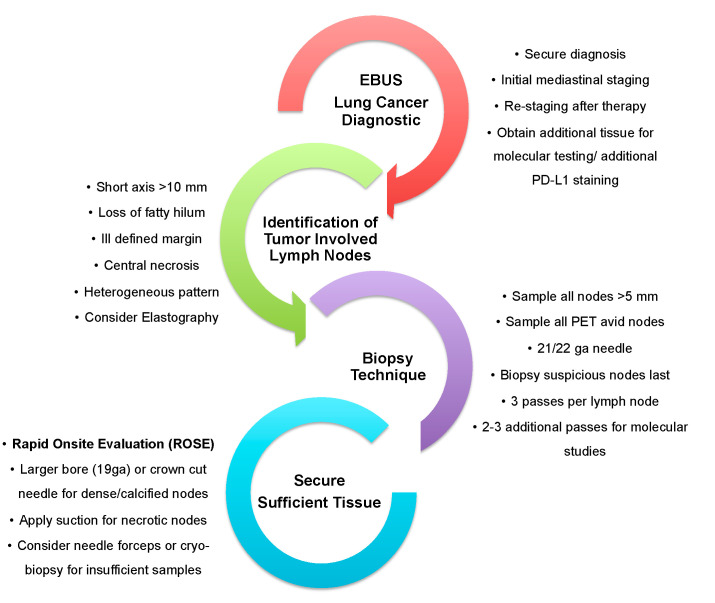
Workflow for endobronchial ultrasound guided precision lung cancer diagnostics.

Thoughtful procedural planning and the flexibility to adapt available biopsy techniques is needed to satisfy increasing tissue demands for EBUS-TBNA. A suggested workflow is illustrated in Figure 2.

### 3.1. Needle Choice

The needle choice is the next important technical consideration because it may affect quality of the EBUS-TBNA biopsy samples. EBUS needles are now available from several manufacturers (Olympus, Boston Scientific, Cook Medical, and Medi-Globe), and they are also available in various sizes (Table 1). The earlier models were composed of stainless steel, and more recently, they are being manufactured using cobalt–chromium and nickel–titanium (nitinol) alloys to provide more flexibility. One of the most recent innovations is a 3-point needle tip design with a crown cut that is used in the Sonotip Topgain^®^ needle (Medi-Globe) and in the Acquire Pulmonary^®^ needle (Boston Scientific, Marlborough, MA, USA). All other needle types have a rounded or beveled single-point needle tip.

The needle size and number of needle passes are both related factors that may determine the tissue yield of the EBUS-TBNA biopsies. Current recommendations of three needle passes to be performed for optimal diagnostic yield were derived from a study by Lee at al. published in 2008, which used the 22G EBUS-TBNA needle from Olympus. At that time, the main focus of EBUS-TBNAs was to provide the best possible diagnostic yield when comparisons to the previously established mediastinoscopy were underway and when molecular testing was still in its infancy [6,26]. In the last couple of years, multiple studies have been conducted to compare needles of various sizes (21G vs. 22G; 21G vs. 19G and 22G vs. 19G needles), all of which resulted in similar diagnostic yields and indicated no advantage regarding diagnostic yields with the use of a larger needle size [27,28,29,30,31,32]. Even though larger needles are gaining popularity, 21G and 22G EBUS needles remain the most widely used due the lack of evidence for an improved diagnostic yield, the necessity of a 2.2 mm working channel to avoid bronchoscope damage, and the increased cost associated with 19G needles.

The CHEST guidelines from 2016 recommended a minimum of three needle passes with a 21G or 22G needle and additional samples for molecular analysis. However, as demonstrated by Ortaköylü and colleagues, more than 40% of EBUS-TBNA samples may be insufficient for pathological work up [20]. The tissue quantity needed for molecular testing has since increased, as PD-L1 staining has become an integrated part of EBUS-TBNA pathological analysis. Because the majority of patients with NSCLC and SCLC are diagnosed at an advanced disease stage with lymph node disease, pre-treatment EBUS tissue samples often remain the most easily accessible samples for molecular testing prior to starting treatment [33]. Therefore, multiple aspects should be considered when choosing the needle size and when optimizing EBUS-TBNA tissue yield for molecular testing. The amount of material obtained with a single pass differs from lymph node to lymph node and between diseases. In some tumor-involved lymph nodes, especially those of poorly differentiated tumors, necrosis can be found in the metastatic lymph nodes and can lead to a liquified consistency, which results in less material acquisition. Patients with a history of sarcoidosis and other granulomatous diseases or coal worker’s pneumonitis tend to have lymph nodes of harder consistencies, which limit the penetration capacity of the needle and result in poor sample acquisition. In our experience, larger bore EBUS needles may be helpful i to secure adequate tissue in these cases. In a recent trial comparing 19G and 22G needles, we used a cut-off of 6 mg sample material measured with a precision balance (Sartorius BP61, Goettingen, Germany); this size was chosen because at this size, the samples were sufficient for pathological analyses (data not shown) and provided at least 40 ng DNA [31,34]. Even though the use of a precision balance might not be feasible in every bronchoscopy suite, the amount of lymphocytes and tumor cells are key for pathological and molecular work-up, including PD-L1 staining. In bronchoscopy suite settings with available rapid on-site evaluation (ROSE) by a cytologist or pathologist, tumor cell amounts and lymphocyte amounts can be estimated on-site and may be used as guidance. The two most recent studies by Pickering et al. and our group have shown that the usage of a 19G EBUS needle generally provides more cells than 22G and 21G needles do [31,33]. Robust data on sample acquisition with the newer Sonotip Topgain needle (Medi-Globe) however is lacking and should be studied further.

A recently introduced technique using transbronchial mediastinal cryobiopsy using a channel created with a TBNA needle, similar to the previously described needle forceps technique, may be used in selected patients to yield additional tissue [35].

### 3.2. Suction or No Suction

Current guidelines leave the decision of whether suction should be applied during EBUS-TBNA sampling up to the bronchoscopist. While the use of suction may cause bleeding and potentially increased tissue damage, it may also result in the aspiration of more cells, particularly in lymph nodes with liquified consistencies. The existing evidence on the benefits of suction are limited to studies that are focused on the ability to secure the diagnosis as opposed to tissue yield [36,37]. Casal et al. conducted the only prospective comparison and found that suction did not improve the diagnostic yield. The quantity of the material obtained with suction compared to the amount of material obtained without suction was not discussed [36]. In a 178-patient retrospective analysis of patients sampled with either a 20-mL VacLok syringe or a 30-mL VacLok syringe using a 22G EBUS needle (Olympus, Vizishot 1), a higher tissue area was reported when the 30-mL VacLok was used. However, the actual negative pressure achieved by either syringe was not reported [37]. Although it is difficult to discern if the vacuum persists throughout all EBUS needle passes, these results suggest that more tissue might be obtained using suction compared to no suction and that the amount of suction that is applied is of importance. Further studies to study the role of suction regarding the cell quantities of lymphocytes and tumor cells are needed. Suction might be beneficial, especially in liquified lymph nodes, to obtain sufficient material for pathological and molecular analysis; however, the use of suction might result in bloodier samples.

## 4. Rapid On-Site Evaluation

A further technical consideration that may affect the quality of EBUS-TBNA samples is the use of a rapid on-site evaluation (ROSE). The first randomized study to test the impact of ROSE on the diagnostic yield of EBUS-TBNAs was performed by Oki and colleagues. A total of 108 patients receiving EBUS-TBNA for suspected lung cancer were randomized for the additional use of ROSE during their EBUS procedure. Since a sample-size calculation had not been conducted and since the difference between the diagnostic yields of the two groups was small (sensitivity: 88% versus 86%), the authors admitted that this study was underpowered [38]. Furthermore, this study was performed before the era of molecular analysis and PD-L1 staining; if ROSE is of advantage for any of these, it has not been tested [38].

Most larger studies on this topic have been retrospective in nature. Nakajima and colleagues analyzed 965 lymph node samples of 438 patients obtained via EBUS-TBNA. Eighty-four lymph nodes (8.7%) were determined to be insufficient for definitive diagnosis by final cytologic evaluation, the non-diagnostic sampling rate was 4.0%, and the rate of false-negative ROSE results was 5.7%. The concordance rate for staging between ROSE and final pathologic diagnosis was 94.3% [39]. Another study published a few months later by Joseph and colleagues analyzed the EBUS-TBNA samples of 131 patients and found that 22 out of 30 samples that were inadequate for ROSE still provided sufficient material for diagnostic purposes [40]. Hence, the authors concluded that the use of ROSE for EBUS-procedures was not beneficial.

Choi et al. demonstrated that the adequacy of ROSE from EBUS-TBNA samples could be significantly increased when an algorithm with the following four criteria was implemented: (1) ≥3 punctures per lymph node, (2) the presence of tumor cells, (3) the presence of microscopic anthracotic pigments (MAP), and (4) lymph node (LD) density of ≥40 cells/field (40× magnification, mean of 10 fields) in smears. On permanent tissue histology, a core tissue size ≥ 2 cm, MAP, and LD ≥ 40 cells/field in smears were significantly related to adequate results. The diagnostic sensitivity of the specimen was increased from 64.7% using a core size ≥ 2 cm as the only criterion to 97% using all four criteria, while the lymph node density >40 cells/field only increased the adequacy by 2% (95% compared to 97%) [41]. The study by Stevenson et al. a showed an increase in diagnostic yield within the first six years of seven years of ROSE practice [42]. This is supported by higher concordance rates between ROSE and final diagnosis reported by Caupena and colleagues (96.1%) in 637 lymph node samples obtained via EBUS-TBNA during that year compared to older publications [43].

More recently, the impact of ROSE on molecular workup has been demonstrated in several studies. In addition to the qualitative assessment for establishing cytologic diagnostic, ROSE may also allow for a quantitative evaluation of the percentage of tumor cells in the smear to gauge suitability for molecular testing. As such, Yarmus and colleagues reported that 95.3% of EBUS-TBNA samples were sufficient for KRAS, EGFR by polymerase chain reaction (PCR), and/or ALK mutation analyses via fluorescence in situ hybridazition (FISH) when four passes were sampled with a 21G needle in the presence of ROSE [44]. Trisolini and colleagues then published the results of a randomized clinical trial, which randomized 126 patients with suspected lung cancer to EBUS with and without ROSE to determine the effect of ROSE on genotyping, which included *EGFR* and *KRAS* testing by next-generation sequencing (NGS), followed by *ALK* testing (FISH) for *EGFR* and *KRAS* wild-type disease. ROSE was associated with a higher percentage of patients for whom the institution’s clinical protocol for lung cancer genotyping was completed (90.8% vs. 80.3%, *p* = 0.09) and a decrease in the number of samples that could only be used for diagnostic purposes (0 vs. 6, *p* = 0.05) [45]. These data show the usefulness of ROSE in confirming the adequacy of tumor cells in EBUS-TBNA samples in order to prevent the need for additional biopsies when molecular analyses are needed.

## 5. Comprehensive Molecular Testing

In the current era of personalized pulmonary oncology and immunotherapies, the necessary amount of tumor DNA and lymphocytes for thorough evaluation via next-generation sequencing (NGS) to detect multiple genes and PD-L1 immunostaining have likely increased beyond what is typically achieved with four sample passes with a 21G needle. The input DNA requirement for NGS analysis varies by testing platform but ranges between 10 ng for Ion Torrent PGM (Life Technologies, Carlsbad, CA, USA) for the detection of 50 genes and 250 ng for Illumina MiSeq (Illumina, San Diego, CA, USA) platforms for the detection of 48 genes [46]. Depending on the target capture method, hybrid versus amplicon-based methods, and the platform type, this translates to about ~100–1000 cells for Ion Torrent and 5000–15,000 cells for Illumina MiSeq [47]. Sufficient tissue yield for NGS may be possible with smaller needles when the number of passes increases. In a retrospective study of 54 EBUS-TBNA samples, Stoy and colleagues showed a high rate for NGS testing for a modified SeqCap EZ (Roche/Nimblegen, Madison, WI, USA), which was successful in 98% of EBUS-TBNA samples for their smaller 50 gene OncoScreen and 91% for the comprehensive 1213 gene OncoPlus Panel [48]. While the authors found no difference in the success rate for NGS between the two small needles, an average of more than six passes was required for both nettle types. Similarly, Casiado and colleagues showed that NGS has a 93.7% success rate during EBUS-TBNA using their 22-gene Oncomine Solid Tumor panel (ThermoFisher, Waltham, MA, USA) [49]. However, the failure rate of the NGS samples was significantly higher from cytoblocks compared to that of smears (9.4 vs. 4.8%) due to lower cellularity and extracted DNA per volume [50]. Our group was able to show that 40 ng of DNA from two EBUS-TBNA passes sampled with a 22G needle were sufficient for an 11-gene NGS panel using the Illumina MiSeq [35]. We also found that significantly more tissue and tumor cells may be obtained with a 19G needle compared to a 22G needle in a randomized trial [31]. The DNA yield of lymph node material obtained with the larger 19G needle (Olympus) or the Sonotip Topgain (Medi-Globe) needle has not yet been reported but may very likely be higher.

## 6. PD-L1 Staining

Immunohistochemical staining with anti-PD-L1 antibodies has become an integral part of EBUS-TBNA pathology workup in lung cancer patients. Single-agent immunotherapy with the anti-PD-1 drug pembrolizumab has been approved by the FDA for the front-line treatment of patients with advanced EGFR/ALK wild-type NSCLC whose tumors have ≥50% tumor-cell PD-L1 expression using the 22C3 test (pharmDx; Dako North America). At advanced stages of NSCLC, EBUS-TBNA material is oftentimes the only available tissue for PD-L-1 staining. Sakakibara and colleagues were able to show good concordance of PD-L1 staining from EBUS-TBNAs with the primary tumor samples and surgically resected metastatic lymph nodes [51]. The concordance rate of the percentage of PD-L1 positive tumor cells from the EBUS-TBNAs obtained from lymph nodes compared to the surgically resected primary tumor were higher when the cut-off was set at ≥1% (87%) then when the cut-off was set to ≥50% when sampled with a 22G needle [52]. EBUS-TBNA material sampled with a 22G needle is not always sufficient for PD-L1 staining, even in the presence of ROSE [52,53]. Yoshimura and colleagues recently reported adequate material in all 67 EBUS-TBNAs sampled with either a 21G or 22G needle and when using more than three needle passes [54]. With regard to the fixation method, Gosney and colleagues demonstrated no significant difference in the interpretation of the PD-L1 staining of EBUS samples fixed with either alcohol-based fixatives compared to those fixed in neutral buffered formalin [55].

## 7. Future Directives

While EBUS-TBNA has been established and widely adopted in clinical practice, there is an ongoing need for high-quality research. Randomized-controlled prospective trials are crucial to define the benefits of technical advancements such as navigational techniques, new robotic platforms, needle designs, and new refinements in cytopathological processing. Study designs need to reflect the increasing demands of lung cancer precision diagnostics by shifting the primary focus from diagnostic yield to cellular quality and quantity. Implementation research will help to further enhance procedural techniques and lead to the widespread adoption of best practices.

## 8. Conclusions

The role of EBUS-TBNA in the management of lung cancer has evolved from a primarily diagnostic and staging modality to also serve as an effective vehicle for molecular testing for the growing number of actionable genetic alterations and for the testing of diagnostic and/or predictive biomarkers. It enables concurrent diagnosis, staging, and molecular analysis, speeding up therapeutic decision-making. The lymphocyte and tumor cell amounts of EBUS-TBNAs matter for molecular work-up and PD-L1 staining can best be estimated when ROSE is used. Because NGS panels differ between institutions, the amount of required DNA varies as well. In general, an addition two to three sample passes with the use if a 21G needle or using an even larger needle design should be considered to optimize the throughput of EBUS-TBNA samples for comprehensive molecular testing, especially when PD-L1 staining is added. The use of suction may also additionally increase the amount of sampled material. Further studies on EBUS-TBNA are needed to establish universal protocols that maximize the yield of cytology material for molecular workup while maintaining the established reliable diagnostic workflow. 

## Figures and Tables

**Table 1:** EBUS needle specifications.

Manufacturer	Model	Needle Size (Gauge)	Needle Tip Specification	MATERIAL
Olympus	Vizishot 1	21, 22		stainless steel
	Vizishot 2	21, 22		nitinol
	Vizishot 2 Flex	19		nitinol
Cook Medical	Echotip	22, 25		stainless steel
	Echotip Procore HD	22, 25		nitinol
Boston Scientific	Expect Pulmonary	22, 25		Cobalt–chromium
	Acquire Pulmonary	22, 25		Cobalt–chromium
Medi-Globe	Sonotip EBUS Pro	22		stainless steel
	Sonotip EBUS Pro Flex	22		nitinol
	Sonotip Topgain	22	3-point needle tip design with a crown cut	nitinol
